# A comparative evaluation between cheiloscopic patterns and the permanent molar relationships to predict the future malocclusions

**DOI:** 10.4317/jced.55776

**Published:** 2019-06-01

**Authors:** Vignesh Ravindra, C. Vishnu Rekha, Sankar Annamalai, D. Ditto Sharmin, Parisa Norouzi-Baghkomeh

**Affiliations:** 1 MDS. Senior Lecturer. Department of Pedodontics and Preventive Dentistry. Saveetha Dental College and Hospital. Chennai; 2 MDS, Professor and Head of the department, Department of Pediatric and Preventive Dentistry, Sathyabama Dental College and Hospital, Chennai; 3 MDS, Reader, Department of Pediatric and Preventive Dentistry, Meenakshi Ammal Dental College and Hospital, Chennai

## Abstract

**Background:**

To assess the correlation between different cheiloscopic patterns with the permanent molar relationships.

**Material and Methods:**

300 children who are 14-16 years old with completely erupted 2nd permanent molars upto occlusal table were recruited and the pattern of molar terminal plane was recorded in the proforma. Lip prints of these subjects were recorded with lipstick-cellophane method and middle 10mm of lower lip was analysed for the lip print pattern as suggested by Sivapathasundharam et al. The pattern were classified based on Tsuchihashi and Suzuki classification.

**Results:**

Type II (branched) pattern was the most predominant cheiloscopic pattern. The predominant patterns which related to the Angle’s classification were; type I (complete vertical) pattern for class I, type IV (reticular) pattern for class II and presence of type IV (reticular) pattern and absence of type I’ (incomplete vertical) pattern for class III. In class III molar relationship, males showed an increased type II (branched) pattern and females showed an increased type IV (reticluar) pattern.

**Conclusions:**

Lip prints can provide an alternative to dermatoglyphics to predict malocclusions in permanent dentition. Further studies with larger sample size are required to provide an insight into its significant correlations.

** Key words:**Cheiloscopy, Angle’s classification, malocclusion.

## Introduction

Oral cavity plays a vital role in functions like mastication, aesthetics, phonetics, communication and emotional expressions etc (1). Well aligned teeth contribute to the health of the oral cavity and stomatognathic system thereby influencing the personality of the individual (2). Malocclusion means that the teeth are in abnormal position in relationship to the basal bone of the alveolar process, to the adjacent teeth and/or to the opposing teeth (3), which compromises the health of oral tissues and also can lead to psychological and social problems (2). Malocclusion is prevalent in varied percentages in various ethnic and racial groups (4-8). There are different methods available to predict malocclusions, which can help a pediatric dentist to attempt any necessary preventive and interceptive orthodontic therapies. Recent studies have focused on linking malocclusions to lip prints (9-11), also in primary dentition (12,13), so that it can help in predicting such conditions. The word “Cheiloscopy” is derived from the Greek word cheilos, which means lips. It is the forensic investigation technique that deals with identification of humans based on lip traces (14). So this study was aimed to assess the correlation between different cheiloscopic patterns with the Angle’s classification of molar relationships.

## Material and Methods

The present study was conducted among a total of 300 children aged 14-16 years attending the Department of Paediatric and Preventive Dentistry. Ethical clearance was obtained from Institutional Review Board. The purpose and procedures of the study were explained to the parents/guardians and informed consent was obtained to participate in the study. Inclusion criteria was children with complete permanent dentition except 3rd molars, with complete occlusal development. Exclusion criteria were previous history of orthodontic treatment, retained deciduous teeth or root stumps, previous history of burn or chemical injury or lesions on lips, different molar relationships on either side of the same subject and uncooperative children. Two calibrated examiners were trained to assess the molar relationships based on the classification given by Angle (1899) (15) as class I, class II, class III. Examination was done using a mouth mirror and recorded in the proforma. Under each molar relationship, 100 children were taken so as to standardize the number of children under each group thereby the results can be closely related to the patterns obtained.

**Figure 1:**
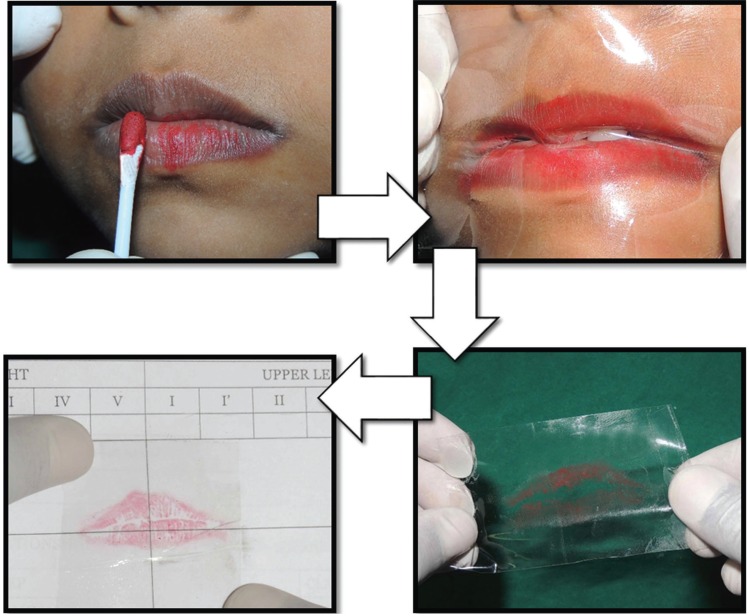
Method for obtaining lip print.

Lip print was recorded by the method proposed by Sivapathasundaram *et al.* (16) which is the lipstick-cellophane technique that provides good clarity and accuracy (17). The lips of the children were cleaned using wet cotton and allowed to dry. Matte finish lip stick was applied with disposable cotton buds as suggested by Amith *et al.* (18). Children were asked to rub their lips gently against one another and then to keep their lips in rest position. The glue part of the cellophane sheet is placed over the lips. After few seconds, the cellophane sheet with lip print was carefully removed and was stuck on to a bonded white paper (Fig. 1). Lip print was checked for clarity and if any smudging of the print was noticed, the procedure was repeated once again. Children were asked to wipe off the remnant lip stick using wet tissue paper.

The collected lip prints were analysed using a magnifying glass by a forensic specialist. The analyst read the lip prints based on the classification given by Suzuki and Tsuchihashi in 1971 (19).

Type I – Complete vertical

Type I’ – Incomplete vertical

Type II - Branched

Type III - Intersected

Type IV - Reticular

Type V – Undetermined or irregular

The middle part of the lower lip (10 mm wide) was taken as study area, similar to the study by Sivapathasundharam *et al.* (16). Lip print pattern was determined by counting highest number of patterns in the above mentioned region.

Statistical analysis

The data values were tabulated and subjected to statistical analysis. For comparison of proportions between all the groups and also between genders, Chi-Square test was applied. Fisher’s exact test was used when any expected cell frequency of less than five were obtained. SPSS version 22.0 was used to analyse the data. A *p*-value of <0.05 is considered as statistically significant.

## Results

The mean age of the children was 15.31 ± 0.67 years. Among the children having class I, 70% were males and 30% were females. For the children having class II, 45% were males and 55% were females. In children having class III, 49% were males and 51% were females. Type II (branched) pattern is the most predominant cheiloscopic pattern which was equally distributed among all the children. An increase in type I (complete vertical) pattern was seen in children with class I molar relation. Type IV (reticular) pattern was seen in higher frequency in class II molar relation. Among children with class III molar relation, presence of type IV (reticular) pattern and absence of type I’ (incomplete vertical) pattern was predominantly seen ( Table 1) There was no statistically significant relationship seen in all three molar patterns when compared with the cheiloscopic patterns (*p* = 0.072). On comparing between genders, among children with class III molar relationship, males showed an increased type II pattern and females showed an increased type IV pattern, which were statistically significant (*p*<0.001) ( Table 2).

**Table 1:**
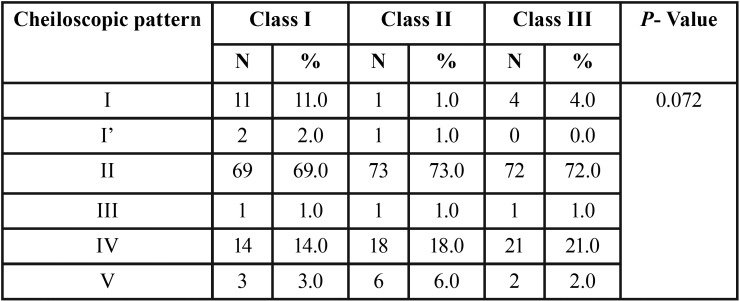
Cheiloscopic distribution in the study subjects.

**Table 2:**
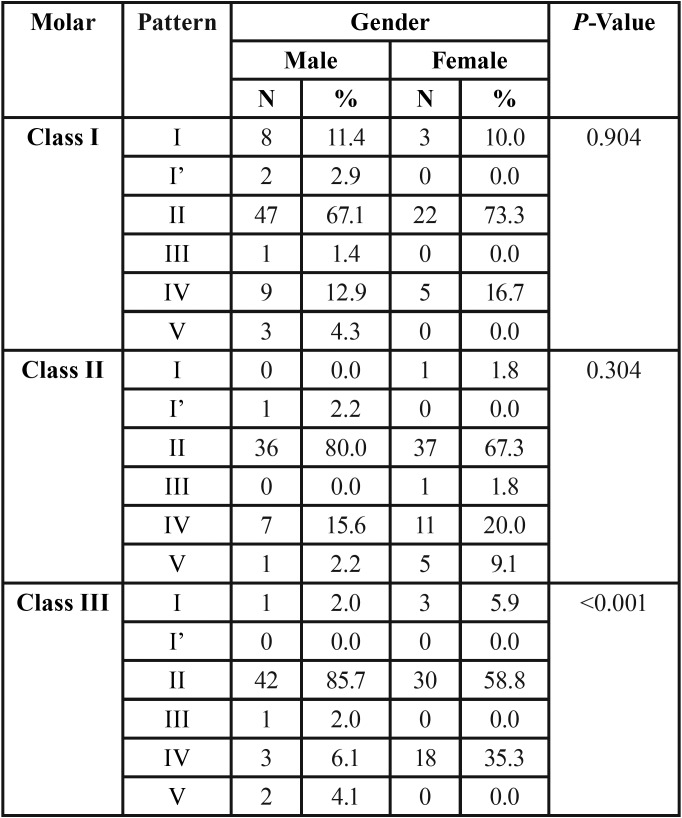
Gender comparison on distribution of cheiloscopic pattern in the study subjects.

## Discussion

The development of occlusion is a result of the interaction and synergistic effects of genetic and environmental factors. The effect of a particular environmental factor on phenotype varies depending on genetic background, which ultimately determines facial and dental morphology (20). Study of lip prints gained importance as early as 1902, which were considered analogous to dermatoglyphics as they remain constant since the time they are formed, unless affected by burns or any pathology. The development of lip, alveolus and palate occurs at the same period i.e. 24th week of intrauterine life and also from the same embryonic origin. Any factor that tends to affect the development of a particular structure will ultimately affect all the other structures that develops along with it. So there is a possibility for the developmental changes that occur in relation to alveolus might be reflected in the cheiloscopic patterns. This was the basis of analysing the permanent molar relationships with the different cheiloscopic patterns.

Literature search showed that there are studies analysing the cheiloscopic pattern and skeletal malocclusions and no studies relating to malocclusion based on Angle’s classification. This study is an attempt to relate them thereby helping the practitioner to predict them. Such a prediction can be helpful to provide preventive and interceptive orthodontic treatment when necessary. The results of the current study has helped us to correlate certain patterns which might be related to specific molar relationships. In our study, among cheiloscopic patterns, type II (branched) pattern was predominant among all the subjects. This was in accordance to the study done by Raghav *et al.* (9) and Madhusudan *et al.* (21), who reported the same predominance in subjects with complete permanent dentition.

In the present study, none of the cheiloscopic patterns showed a significant relationship for permanent molar relationships. This was contradicting to the study done by Raghav *et al.* (9) who had reported type II pattern among skeletal class I individuals and type I pattern among skeletal class III individuals. The results obtained could not be correlated with the study done by Kulkarni *et al.* (10) as the lip prints were analysed from all the quadrants of the lips contradicting to the current study which involves only middle 10mm of lower lip.

The results of the present study showed the predominant patterns which related to the different molar relations were; type I (complete vertical) pattern for class I, type IV (reticular) pattern for class II and presence of type IV (reticular) pattern and absence of type I’ (incomplete vertical) pattern for class III. In class III molar relationship, males showed an increased type II (branched) pattern and females showed an increased type IV (reticluar) pattern

One of the limitations of the study is that this study covers genetic factors only, though environmental and local factors also plays significant role in determining malocclusion (22). The threshold theory as has been advanced by independent studies conducted by Carter and Matsunaga implies that only when the combined factors exceed a certain level, can these abnormalities be expected to appear. The aetiological factors responsible for the manifestation of cheloscopic patterns and malocclusion might not cross this threshold for these conditions to manifest clinically (23). The other limitation is that further studies with larger sample size involving multiple ethnic groups are required to provide a more accurate prediction.

These results could help the dental practitioner to establish necessary measures during the primary and mixed dentition period itself so as to ensure no loss of space occurs due to reasons of dental caries or premature extraction of primary teeth.

## Conclusions

Within the limitations of the current study, cheiloscopy can serve as a non-invasive aiding tool in predicting the molar relations that can help in preventive orthodontic management but larger samples with different ethnic origins are needed to support these findings.
